# Health literacy and its association with mental and spiritual well-being among women experiencing homelessness

**DOI:** 10.1093/heapro/daae019

**Published:** 2024-03-02

**Authors:** Andreas Karlsson Rosenblad, Anna Klarare, Penny Rapaport, Elisabet Mattsson, Sophie Nadia Gaber, in collaboration with the Women’s Advisory Board for Inclusion Health

**Affiliations:** Department of Statistics, Uppsala University, Box 513, 751 20, Uppsala, Sweden; Department of Medical Sciences, Division of Clinical Diabetology and Metabolism, Uppsala University, Akademiska sjukhuset, 751 85, Uppsala, Sweden; Department of Neurobiology, Care Sciences and Society, Division of Family Medicine and Primary Care, Karolinska Institutet, 171 77, Stockholm, Sweden; Department of Women’s and Children’s Health, Healthcare Services and e-Health, Uppsala University, Akademiska sjukhuset, 751 85, Uppsala, Sweden; Department of Healthcare Sciences, Marie Cederschiöld University, Box 11189, 100 61, Stockholm, Sweden; Division of Psychiatry, Faculty of Brain Sciences, University College London, Maple House, W1T 7BN, London, UK; Department of Women’s and Children’s Health, Healthcare Services and e-Health, Uppsala University, Akademiska sjukhuset, 751 85, Uppsala, Sweden; Department of Healthcare Sciences, Marie Cederschiöld University, Box 11189, 100 61, Stockholm, Sweden; Department of Women’s and Children’s Health, Healthcare Services and e-Health, Uppsala University, Akademiska sjukhuset, 751 85, Uppsala, Sweden; Department of Healthcare Sciences, Marie Cederschiöld University, Box 11189, 100 61, Stockholm, Sweden; Department of Healthcare Sciences, Marie Cederschiöld University, Box 11189, 100 61, Stockholm, Sweden

**Keywords:** health literacy, homelessness, psychological distress, spirituality, psychological well-being, women’s health

## Abstract

Low health literacy (HL) has been linked to low self-rated health, reduced efficacy of behaviour change, and challenges in preventing, treating, or managing health conditions. People experiencing homelessness are at risk of poor HL; however, few studies have investigated HL in relation to mental and spiritual well-being among people experiencing homelessness in general, or women experiencing homelessness specifically. This cross-sectional study of 46 women experiencing homelessness in Stockholm, Sweden, recruited during the period October 2019–December 2020, aimed to examine how HL was associated with mental and spiritual well-being among women experiencing homelessness. Participants answered questions about socio-demographic characteristics (age, length of homelessness, education) and digital technology (mobile phone/the Internet) use, in addition to Swedish language versions of three questionnaires administered through structured, face-to-face interviews: the Communicative and Critical Health Literacy Scale, the General Health Questionnaire 12 and the Functional Assessment of Chronic Illness Therapy-Spiritual Well-Being. Data were analysed using linear regression, which revealed statistically significant associations between HL and mental well-being (*p* = .009), and between HL and spiritual well-being (*p* = .022). However, neither socio-demographic characteristics nor digital technology use were significantly associated with HL. In conclusion, promoting HL may improve mental and spiritual well-being in this vulnerable population. An advisory board of women with lived experiences of homelessness (*n* = 5) supported the interpretation of the findings and emphasised the need to consider HL in relation to basic needs such as ‘housing first’. Moreover, health information and services should be accessible to people with different degrees of HL.

**Contribution to Health Promotion**

- This study is among the first to examine health literacy (HL) in relation to mental and spiritual well-being among women experiencing homelessness.
- A higher degree of HL was associated with decreased psychological distress and enhanced spiritual well-being among women experiencing homelessness.
- The findings highlight the importance of considering HL in health promotion interventions for women experiencing homelessness, who are disproportionately impacted by the burden of disease and disability compared to the general population.

## BACKGROUND

Health literacy (HL), the capacity to obtain, understand and use health-related information to make appropriate health decisions, has been suggested to be a precondition and indicator of ensuring healthy lives and promoting well-being, in accordance with the 2030 Agenda for Sustainable Development (Christie and Ratzan, 2020; Liu *et al*., 2020). People experiencing homelessness are at risk of poor HL (Odoh *et al*., 2019; Radó *et al*., 2022). Poor HL has been linked to low self-rated health (Furuya *et al*., 2015; Marques *et al*., 2018), reduced efficacy of behaviour change (Lee, 2017; Odoh *et al*., 2019), and challenges in preventing, treating or managing health conditions (Lee, 2017; Odoh *et al*., 2019). A systematic review exploring the relationship between HL and health disparities found that most studies focused on racial/ethnic and educational disparities (Mantwill *et al*., 2015). However, less is known about the effects of HL on other marginalised and underserved groups, such as people experiencing homelessness, sex workers, prisoners and people with substance use disorders (SUDs). Women experiencing homelessness are disproportionately affected by the burden of disease and disability (Fazel *et al*., 2014; Tweed *et al*., 2021; Vickery *et al*., 2021), with elevated risks of premature and often preventable mortality (Aldridge *et al*., 2018). Hence, women experiencing homelessness require high levels of care and access to health information and services (Phipps *et al*., 2019; Milaney *et al*., 2020; Kneck *et al*., 2021), and there is a need for research to address the knowledge gap regarding how HL is associated with health and well-being among women experiencing homelessness.

Homelessness has been linked to high rates of mental health comorbidities (Fazel *et al*., 2014; Gutwinski *et al*., 2021), and the incidence of mental health issues, such as anxiety and depression, is higher among women experiencing homelessness than the general population (Phipps *et al*., 2019), or males experiencing homelessness (Chan *et al*., 2023). Adverse childhood experiences (e.g. physical or sexual abuse during childhood) are prevalent among women experiencing homelessness (Milaney *et al*., 2020; Liu *et al*., 2021), and they have an increased risk for traumatic experiences, such as physical or sexual violence while living on the streets or in shelters (Milaney *et al*., 2020). Research among women experiencing homelessness has shown higher rates of SUDs compared to both women with low socioeconomic status and the general population (Upshur *et al*., 2017). Moreover, a Swedish study found that 91% of women with uncertain housing and substance use had been exposed to male violence (Beijer *et al*., 2018). Exposure to violence has a detrimental impact on the health and well-being of women experiencing homelessness, including post-traumatic stress disorder (PTSD), which is linked to excess mortality from suicide, medical causes and other drug-related problems (Ayano *et al*., 2020). Early screening and treatment of mental health conditions such as PTSD may thus help to reduce excess mortality and promote health and well-being among women experiencing homelessness.

While excess mortality and morbidity among people experiencing homelessness are widely reported (Fazel *et al*., 2014; Aldridge *et al*., 2018), there is a knowledge gap regarding other aspects of well-being in this population. A previous study found that people experiencing homelessness had significantly higher spirituality and religiosity compared to those who were housed, and that spirituality and religion were perceived as a coping mechanism for people experiencing homelessness (Ahuja *et al*., 2020). Spiritual well-being includes aspects of faith but also encompasses a broader sense of meaning and purpose in one’s life (Peterman *et al*., 2002, 2014). Spiritual well-being is a component of health-related quality of life (HRQOL) and increased spiritual well-being has been shown to have a positive effect on a person’s quality of life (Munoz *et al*., 2015; Ahmad *et al*., 2022). Studies have highlighted a relationship between spiritual well-being and health promotion, as well as coping mechanisms for various populations, including people with cancer (Damen *et al*., 2021; Kavalali Erdoğan & Koç, 2021), older adults in nursing homes (Santana-Berlanga *et al*., 2020; Rykkje *et al*., 2023) and those at risk of suicide while living with chronic healthcare issues (Loureiro *et al*., 2018).

Women experiencing homelessness have multiple, complex healthcare needs and live in challenging life situations which necessitate access to primary, preventative and specialised healthcare based on gender-sensitive and trauma-informed care (Milaney *et al*., 2020). Despite the prevalence of complex mental health issues among women experiencing homelessness, only a small proportion of this population is engaged in psychiatric or psychological treatment (Phipps *et al*., 2019). Research suggests that women experiencing homelessness face systemic, individual and cultural barriers to accessing appropriate healthcare services, such as a lack of gender-specific or trauma-informed care (Milaney *et al*., 2020), fragmented and uncoordinated services (Lawrie *et al*., 2020; Mar *et al*., 2023), insecure access to digital technology (Heaslip *et al*., 2021a, 2021b) and limited resources to manage competing needs, including long waiting times, lengthy travel distances and lack of transportation (Allen and Vottero, 2020; Milaney *et al*., 2020). Crucially, less is known about the barriers this population experiences in accessing health information and services, which motivates the necessity to develop empirical insights about whether HL is associated with mental health and spiritual well-being among women experiencing homelessness.

### Aim

To examine how HL is associated with mental and spiritual well-being among women experiencing homelessness. It was hypothesised that differences in HL would be associated with differences in mental and spiritual well-being.

## METHODS

### Study design and setting

This cross-sectional study was performed in Stockholm, Sweden, during the years 2019–2020 as part of a larger project to promote inclusion health for women experiencing homelessness. Details of the project, which involved the development of an advisory board of women with lived experiences of homelessness (i.e. The Women’s Advisory Board for Inclusion Health), are provided in previous publications (Kneck *et al*., 2021; Mattsson *et al*., 2023). This study followed the Strengthening the Reporting of Observational Studies in Epidemiology (STROBE) guidelines (von Elm *et al*., 2008).

### Participants

The present study focuses on women experiencing homelessness and is part of a larger study which, besides women experiencing homelessness, also included registered nurses and nursing students. Further details about this larger study, including power calculations specifying that at least 35 women experiencing homelessness should be recruited, are given in previous publications (Gaber *et al*., 2022a, 2022b). Notably, the study sample of 46 women included in the present study was larger than the threshold value of 35 women obtained from the power calculations.

Women experiencing homelessness were recruited from a primary healthcare centre (PHCC) in Stockholm, Sweden, with support from a research assistant who had experience working with the study population. The PHCC is privately run with public funding and provides a variety of free, drop-in and referral, healthcare services for people experiencing homelessness. There are approximately 14,000 annual visits to the PHCC with 40% of the 1,300 people who receive care being women.

A convenience sample of 46 women experiencing homelessness was recruited to the study according to the inclusion criteria that they were Swedish-speaking and with experience of one or more categories of homelessness based on the Typology of Homelessness and Housing Exclusion (ETHOS): (1) rooflessness (e.g. without a shelter of any kind, sleeping rough); (2) houselessness (e.g. with a place to sleep but temporary in institutions or shelters); (3) living in an insecure accommodation (e.g. at risk of severe exclusion for reasons such as insecure tenancies) and (4) living in an inadequate accommodation (e.g. in unfit housing) (European Federation of National Organisations Working with the Homeless [FEANTSA], 2017). We consulted with staff at the recruiting site and based on their expertise and experience with the women, those expressing severe anxiety and/or distress, or demonstrating violent and/or abusive behaviours, were not approached for study inclusion.

### Data collection

The first wave of data collection occurred between October and November 2019. It was temporarily paused during the Coronavirus (COVID-19) pandemic and resumed with a second wave between September and December 2020. Written and oral information was shared with potential participants, who were given the opportunity to discuss the study prior to data collection. Data were collected by structured face-to-face interviews at the PHCC. The research assistant read the questions aloud to the participants and recorded their responses on the written questionnaire. The rationale for administering the questionnaires through interviews was to ensure that women experiencing homelessness, who often have multiple health problems including concentration issues, could fully engage with the study. This approach aimed to facilitate their participation, allowing them to ask for clarification and receive support from the research assistant as needed. Once data collection ended, participants were compensated with a grocery store voucher valued at SEK 100 (approximately €10).

### Instruments

As part of the larger research project, participants answered questionnaires covering attitudes to homelessness, caring behaviours and exposure to violence. However, this study focused on data collected using the Swedish versions of the Communicative and Critical Health Literacy Scale (C & C HLS), General Health Questionnaire 12 (GHQ-12) and the Functional Assessment of Chronic Illness Therapy-Spiritual Well-Being (FACIT-Sp-12) instruments. We also collected socio-demographic information and asked whether the participant was using a mobile phone or the Internet. Education level was measured using five categories (not finished primary school, primary school, secondary school, college/university or other), while length of homelessness was measured using four categories (0–1 year, > 1 year but < 5 years, 5–10 years and > 10 years).

#### The Communicative and Critical Health Literacy Scale (C & C HLS)

HL was measured using the Swedish version of the Japanese Communicative and Critical Health Literacy Scale (C & C HLS) (Ishikawa *et al*., 2008; Wångdahl and Mårtensson, 2014). It consists of five items with responses scored on a 5-point scale ranging from ‘strongly disagree’ (1 point) to ‘strongly agree’ (5 points). The total score range is 5–25 points, with higher scores indicating higher HL. After rescaling the 5-point scale such that a score of 1–2 results in a value of 1000, a score of 3 gives a value of 100, and a score of 4–5 gives a value of 1, the transformed values are summarised and each respondent is categorised as having a ‘Deficient communicative and critical HL’ for sums ≥ 1000, ‘Problematic communicative and critical HL’ for sums > 100 and < 1000, and ‘Adequate communicative and critical HL’ for sums < 100 (Mårtensson and Wångdahl, 2017). The Swedish version of the C & C HLS is valid and reliable for use in the Swedish population, with good internal consistency (Cronbach’s α = 0.87) (Wångdahl and Mårtensson, 2014; Jaensson *et al*., 2021).

#### The General Health Questionnaire 12 (GHQ-12)

The General Health Questionnaire 12 (GHQ-12) instrument was developed as a screening tool for detecting psychological distress and is used for assessing mental well-being by identifying distressing symptoms (Goldberg *et al*., 1997; Comotti *et al*., 2023). It is a validated instrument used in various clinical settings due to its convenience of use and high reliability, sensitivity and specificity (Goldberg *et al*., 1997). The GHQ-12 includes 12 items rated on a 4-point scale, ranging from ‘never’ (1 point) to ‘always’ (4 points); the total score range is 12–48 points, with higher scores corresponding to higher psychological distress. The Swedish version of the GHQ-12 was used in the present study and it has been used extensively in national and regional settings. It has excellent discriminant validity and good internal consistency (Cronbach’s α = 0.94) (Lundin *et al*., 2017). A total GHQ-12 score > 20 is considered common for people experiencing ongoing depression and/or anxiety.

#### *Functional Assessment of Chronic Illness Therapy*–*Spiritual Well-Being 12-Item Scale (FACIT-Sp-12)*

The Functional Assessment of Chronic Illness Therapy–Spiritual Well-Being 12-Item Scale (FACIT-Sp-12) instrument is part of the larger FACIT measurement system and is used extensively to measure spiritual well-being among people with cancer and other chronic illnesses (Peterman *et al*., 2002, 2014; Munoz *et al*., 2015). It has three sub-scales measuring faith, meaning and peace which are aligned with conceptual models of spiritual well-being (Munoz *et al*., 2015). It has been translated into > 40 languages and is used extensively to investigate associations between spiritual well-being, health and adjustment to illness (FACIT Group, 2021). The FACIT-Sp-12 has been used to measure spiritual well-being across different degrees of spirituality and/or religious traditions (including people who identify as spiritual yet not religious) and has acceptable internal consistency (>0.70) for all sub-scales (Munoz *et al*., 2015). Details of the Swedish version of the FACIT-Sp-12, used in the present study, are provided in previous publications (Mårtensson *et al*., 2008). Using the Swedish version of the FACIT-Sp-12, respondents were asked to describe aspects of spirituality and/or faith that contributed to HRQOL during the past 7 days (Munoz *et al*., 2015). The Swedish version of the FACIT-Sp-12 items includes a 5-point Likert scale ranging from ‘not at all’ (0) to ‘very much’ (4). Two items are negatively worded and require reverse coding. A total score and individual sub-scores are calculated, with higher scores indicating higher spiritual well-being.

### Statistical analyses

Categorical data are presented as frequencies and percentages, *n* (%), while ordinal and continuous data are given as means with accompanying standard deviations (SDs). Linear regression models were used to estimate how HL measured with the C & C HLS was associated with both mental well-being measured with the GHQ-12 instrument and spiritual well-being measured with the FACIT-Sp-12 instrument. Additionally, linear regression models were used to estimate how age (years), using a mobile phone, using the Internet, experiencing homelessness for ≥ 5 years and having a college/university education were associated with HL measured with the C & C HLS. The results are presented as slope coefficient β with accompanying 95% confidence intervals (CIs). All statistical analyses were performed using R version 4.2.0 (R Core Team, 2022), with two-sided *p*-values < 0.05 considered statistically significant.

#### The Women’s Advisory Board for Inclusion Health

We consulted with an advisory board of women with lived experiences of homelessness to seek insights into the interpretation of results and to discuss potential implications for both research and practice. In November 2023, five women from the Women’s Advisory Board for Inclusion Health participated in a 2-hour workshop with the goal of enhancing the relevance and applicability of the findings. The workshop comprised brainstorming sessions and individual reflections, aiming to incorporate the experiences of women who have lived through homelessness. The second and fourth authors attended the workshop alongside the women and facilitated the session.

### Ethical considerations

This study was approved by the Regional Ethical Board in Stockholm, Sweden (Number 2019-021130) and adhered to the principles of the Declaration of Helsinki (General Assembly of the World Medical Association, 2014). Written informed consent was obtained from all participants prior to data collection and the participants were also informed of their rights to withdraw from the study at any time without the need to give a reason.

## RESULTS

Characteristics of the study sample of 46 women are given in Table 1. The participating women were at a mean (SD) age of 47.6 (10.4) years, with a majority having an education at the secondary school or college/university level (*n* = 28; 60.9%) and experiencing homelessness for < 5 years (*n* = 25; 54.3%). Most women used a mobile phone (*n* = 38; 82.6%) as well as the Internet (*n* = 37; 80.4%). The mean (SD) C & C HLS total score was 19.0 (3.8) points, with 15 (32.6%) women considered as having an ‘Adequate communicative and critical HL’. The mean (SD) GHQ-12 total score was 19.8 (6.8) points, with 17 (37.0%) women having a total score of ≥ 21 points, indicating ongoing depression and/or anxiety. Finally, the mean (SD) FACIT-Sp-12 total score was 25.6 (10.8) points.

**Table 1:** Characteristics of the study sample of *n* = 46 women experiencing homelessness

Variable	Value	Missing, *n* (%)
Age, mean (SD)	47.6 (10.4)	1 (2.2)
Education level, *n* (%)		0 (0.0)
− Not finished primary school/ Other	4 (8.7)	
− Primary school	14 (30.4)	
− Secondary school	17 (37.0)	
− College/University	11 (23.9)	
Length of homelessness, *n* (%)		0 (0.0)
− 0–1 year^a^	11 (23.9)	
− > 1 year but < 5 years	14 (30.4)	
− 5–10 years	13 (28.3)	
− >10 years	8 (17.4)	
Using a mobile phone, *n* (%)	38 (82.6)	0 (0.0)
Using the internet, *n* (%)	37 (80.4)	0 (0.0)
C & C HL scale total score (points), mean (SD)^b^	19.0 (3.8)	0 (0.0)
− Deficient, *n* (%)	15 (32.6)	
− Problematic, *n* (%)	16 (34.8)	
− Adequate, *n* (%)	15 (32.6)	
GHQ-12 total score (points), mean (SD)^c^	19.8 (6.8)	5 (10.9)
− 0–15 points, *n* (%)	10 (21.7)	
− 16–20 points, *n* (%)	14 (30.4)	
− 21–36 points, *n* (%)	17 (37.0)	
SP-12 total score (points), mean (SD)^d^	25.6 (10.8)	6 (13.0)

Note: SD, standard deviation. ^a^Including one woman stating that she had been experiencing homelessness ‘for periods’. Score range: ^b^5–25 points, ^c^0–36 points, ^d^0–48 points.

### Results from regression models

Table 2 presents the results from unadjusted linear regression models for the outcomes of HL, mental well-being and spiritual well-being, measured with the C & C HLS, GHQ-12 and FACIT-Sp-12 instruments, respectively. Notably, there were no statistically significant associations between any of the examined independent variables (i.e. age, using a mobile phone, using the Internet, experiencing homelessness for ≥ 5 years or having a college/university education) and the HL outcome. There were, however, statistically significant associations between HL and the mental well-being outcome (*p* = 0.009), as well as between HL and the spiritual well-being outcome (*p* = 0.022). Specifically, a higher degree of HL implied lower psychological distress, with a one-point higher C & C HLS total score on average corresponding to a 0.73 points lower GHQ-12 total score. Likewise, a higher degree of HL implied higher spiritual well-being, with a one-point higher C & C HLS total score on average giving a 1.06 points higher FACIT-Sp-12 total score. However, none of the independent variables (i.e. age, using a mobile phone, using the Internet, experiencing homelessness for ≥ 5 years or having a college/university education) were significantly associated with mental or spiritual well-being.

**Table 2:** Results from unadjusted linear regression models for the outcomes C & C HL scale, GHQ-12 and SP-12

Outcome	Variable	β	95% CI	*p*-value	*R* ^2^ (%)
C & C HL scale	Age (years)	0.08	−0.02; 0.19	0.118	5.58
	Using a mobile phone	−0.15	−3.13; 2.82	0.919	0.02
	Using the internet	0.55	−2.29; 3.39	0.697	0.35
	Experiencing homelessness ≥ 5 years	1.23	−1.01; 3.46	0.274	2.71
	College/University	1.19	−1.42; 3.81	0.363	1.88
GHQ-12	Age (years)	−0.11	−0.32; 0.10	0.302	2.73
	Using a mobile phone	−0.20	−6.38; 5.98	0.948	0.01
	Using the internet	−1.07	−6.86; 4.73	0.711	0.35
	Experiencing homelessness ≥ 5 years	0.32	−4.06; 4.70	0.884	0.06
	College/University	−2.68	−7.69; 2.33	0.285	2.92
	C & C HL scale (score)	−0.73	−1.27; −0.19	**0.009**	16.06
SP-12	Age (years)	0.22	−0.13; 0.57	0.220	3.94
	Using a mobile phone	−5.38	−14.44; 3.67	0.236	3.68
	Using the internet	−3.36	−12.05; 5.33	0.439	1.59
	Experiencing homelessness ≥ 5 years	0.53	−6.48; 7.54	0.879	0.06
	College/University	2.22	−6.51; 10.95	0.610	0.69
	C & C HL scale (score)	1.06	0.16; 1.96	**0.022**	12.96

Note: CI, confidence interval; SD, standard deviation. Significant *p*-values are given in **bold**.

## DISCUSSION

The present study of how HL was associated with mental and spiritual well-being in a sample of 46 women experiencing homelessness in Stockholm, Sweden, found that higher degrees of HL implied lower psychological distress and higher spiritual well-being.

### Results in perspective

#### Health literacy and psychological distress among women experiencing homelessness

The finding of the present study that a higher degree of HL was associated with lower psychological distress among women experiencing homelessness is important since women experiencing homelessness are known to have high rates of mental health comorbidities (Fazel *et al*., 2014; Gutwinski *et al*., 2021). This finding substantiates earlier research that revealed a positive association between HL and self-rated health among people experiencing homelessness (Odoh *et al*., 2019). The present study contributes novel insights about the impact of HL on psychological distress from the perspective of women experiencing homelessness. This differs from prior studies which have tended to focus on aggregated samples of people experiencing homelessness (Odoh *et al*., 2019), or the link between HL and more general measures of self-rated health among housed populations (Aaby *et al*., 2017; Storey *et al*., 2020; Dolezel and Hewitt, 2023). These prior studies are not generalisable to women experiencing homelessness, but similar to our findings, the studies showed positive associations between HL and self-rated health (Aaby *et al*., 2017; Storey *et al*., 2020; Dolezel and Hewitt, 2023).

#### Health literacy and spiritual well-being among women experiencing homelessness

To the author’s knowledge, the present study is the first to investigate the association between HL and spiritual well-being among women experiencing homelessness. Thus, the finding that a higher degree of HL was associated with higher spiritual well-being contributes a novel insight into the potential role of HL in broader aspects of health promotion, such as spiritual well-being. One could thus infer that spiritual well-being may be promoted through enhanced HL among women experiencing homelessness. Earlier research suggests that spirituality and religious beliefs can impact individuals’ mental and physical health, and yet there is a lack of professional knowledge and there are organisational barriers to the provision of spiritual care by healthcare professionals (Harorani *et al*., 2022). A more holistic understanding of spiritual well-being in relation to health is needed among healthcare professionals to enable them to promote spiritual well-being among their patients, including women experiencing homelessness.

Together with earlier research (Ahuja *et al*., 2020), the findings suggest that there is a need to promote spiritual well-being, since people experiencing homelessness have been found to perceive spirituality and religiosity as important coping mechanisms in their everyday life. One of the few studies to explore spiritual well-being among people experiencing homelessness found that religiousness and spirituality were important factors in the lives of men and women experiencing homelessness, and both religiousness and spirituality were associated with lower suicidal ideation (Vitorino *et al*., 2021). A few studies have explored the association between HL and spiritual well-being in different populations, such as maternal HL among women (Khorasani *et al*., 2022), or patients living with kidney disease (undergoing haemodialysis) (Hassani *et al*., 2022). Moreover, earlier research has emphasised the need to increase social support to promote spiritual issues, specifically, the meaning of life among women with breast cancer (Jadidi and Ameri, 2022). However, the findings from these earlier studies are inconsistent and not generalisable to women experiencing homelessness. Hence, further empirical research among women experiencing homelessness is needed. The findings of the present study point to the need for future qualitative research to facilitate a deeper understanding of the association between HL and spiritual well-being among women experiencing homelessness.

#### Health literacy, sociodemographic characteristics and digital technology use among women experiencing homelessness

The present study found no statistically significant associations between any of the examined independent variables (i.e. age, using a mobile phone, using the Internet, experiencing homelessness for ≥ 5 years or having a college/university education) and the HL outcome. These findings contradict earlier research which suggests that variables such as age (Dolezel and Hewitt, 2023), education (Stormacq *et al*., 2019) and access to digital technology (e.g. mobile phones and the Internet) (Bailey *et al*., 2015) may impact HL among housed populations. The lack of a statistically significant association between education and HL also differs from an earlier study among people experiencing homelessness or who were vulnerably housed with mental health disorders, which found that higher levels of education were associated with higher levels of HL (Farrell *et al*., 2020). The findings from the present study may be contextualised according to a previous cross-national survey in eight European countries, including Sweden, which cautioned against linking homelessness to lower education levels (Taylor *et al*., 2019).

The present study’s finding that the majority of women experiencing homelessness reported using a mobile phone (82.6%) and the Internet (80.4%) is consistent with a systematic review that estimated that 80% of people experiencing homelessness own a mobile phone or smartphone (Heaslip *et al*., 2021b). Research suggests that while people experiencing homelessness may have access to mobile phones and report enthusiasm for using them to obtain health-related information and services (e.g. appointment or prescription reminders) and to help address issues such as depression, anxiety, self-harm, abuse or substance use (Adkins *et al*., 2017), they face considerable personal (e.g. privacy concerns, fear of theft, distrust about data management) and practical barriers (e.g. lack of charging points, limited data) that inhibit them from using mobile phones to access relevant health-related information and services (Heaslip *et al*., 2021a, 2021b). Although reported use of mobile phones and the Internet was not significantly associated with HL in the present study, earlier research has found literacy-related disparities in access and use of mobile phones and the Internet for housed populations, which may indicate the need for future digital health interventions to ensure that health promotion initiatives do not compound these disparities (Bailey *et al*., 2015).

#### Health promotion strategies among women experiencing homelessness

Earlier research on the social determinants of HL recommends multicomponent interventions to improve HL and health status (Dolezel and Hewitt, 2023). Recommended interventions to promote HL include adult education, support groups, e-health online resources and specifically interventions to improve patient-provider interactions through social and literacy support that enables patients to develop self-efficacy and reinforce healthy behaviours (Dolezel and Hewitt, 2023). While these interventions may prove effective for the general population, the Women’s Advisory Board for Inclusion Health underscored a more nuanced perspective based on their lived experiences. Similar to earlier research (Omerov *et al*., 2020; Paudyal *et al*., 2020), the women emphasised that, when experiencing homelessness, health becomes a secondary concern, with priorities centred on meeting basic needs such as shelter, food, sleep and safety. They expressed that, in such circumstances, body awareness diminishes and conventional strategies to target HL may not resonate.

Aligning with insights from earlier research (Luchenski *et al*., 2018; Kopanitsa *et al*., 2023), the Women’s Advisory Board for Inclusion Health recommended a different approach. They suggested that HL interventions should be integrated into outreach health promotion initiatives—services provided outside traditional healthcare settings and closer to locations frequented by the target population. The women’s perspective highlights the importance of recognising the immediate needs of this marginalised and under-served population, asserting that addressing housing stability should precede health considerations. Thus, a ‘housing first’ approach (i.e. provision of stable housing without imposing conditions on receiving support such as abstinence from substance use) (Baxter *et al*., 2019), together with adequate support, is viewed as a prerequisite for subsequent health-related interventions, according to the women’s experiences.

On the one hand, the findings in the present study are aligned with earlier research emphasising the need to increase HL skills among people experiencing homelessness, to promote their health and well-being (Odoh *et al*., 2019). Odoh *et al*. (2019) proposed a targeted approach to provide special attention and interventions for people experiencing homelessness who have low HL, to support their ability to understand and process health information which may result in improved self-rated health (Odoh *et al*., 2019). Still, adaptations to make health information and services more accessible are needed to remove barriers such as the expectation of high levels of education, since approximately 60% of our participants had education at the secondary school or college/university level. Research shows that people experiencing homelessness are less likely than other groups to receive health education during healthcare encounters and a review highlighted the importance of developing health education interventions that target specific barriers faced by people experiencing homelessness (e.g. difficulties navigating services, fears and misconceptions) (Lawrie *et al*., 2020). On the other hand, interventions targeting people experiencing homelessness risk segregating specific groups of healthcare users. A targeted approach could also exacerbate unintentional biases in healthcare encounters (Lawrie *et al*., 2020) such as shame and stigmatising attitudes towards homelessness, which have been shown to negatively impact the caring behaviours that women experiencing homelessness receive (Gaber *et al*., 2022b). Thus, research increasingly points to a proportionate universalist approach, which proposes that health promotion interventions should be developed and implemented to ensure that they are as accessible and usable as possible for everyone, but that simultaneously offer tailored approaches to reach and engage with groups that are disproportionately impacted by the effects of poor HL, such as women experiencing homelessness (Stormacq *et al*., 2019).

### Strengths and limitations

Among the strengths of the present study is its novel, as it contributes to the discourse on HL in relation to health and well-being from the perspective of women experiencing homelessness. Thus, the results of the present study may act as support for initiatives to investigate HL among populations who may be at risk of marginalisation and health inequalities, but who lack opportunities for involvement in health research, to inform more targeted policies and healthcare deliveries.

However, the findings of the present study should be interpreted carefully, taking into account its limitations. Firstly, the sample size was relatively small, including only 46 women. This meant that associations were only detectable, in terms of statistical significance, for independent variables with a large influence on the outcome. Some of the independent variables that were found to be non-significant in the present study may thus have been significant if a larger sample size had been used. However, despite the small sample size, it was still larger than the threshold value obtained from the power calculations, which suggested that a sample size of 35 women was needed for this hard-to-reach group. Moreover, despite the small sample size, the study revealed some interesting results which may inform further research, and the study is among the first to explore HL among women experiencing homelessness. The relatively small sample size of the study may to a large part be explained by challenges in recruiting and performing research in this hard-to-reach and underserved population, with the data collection period partially coinciding with the global COVID-19 pandemic.

Secondly, the convenience sampling, which occurred in a single PHCC in Stockholm, Sweden potentially limited the size and diversity of the sample, as well as the generalisability of the results. For example, the high education levels among the participants of the present study may be partly attributed to Sweden’s provision of publicly funded higher education for citizens from the European Union and European Economic Area. Moreover, the study potentially did not include women who were very distressed. The mean GHQ-12 score in the present study was slightly below the common threshold for people experiencing ongoing depression and/or anxiety (≥21 points). Nevertheless, 37.0% of the women in the present study had total scores of ≥ 21 points, indicating ongoing depression and/or anxiety, which supports earlier research suggesting that women experiencing homelessness are at risk of depression and anxiety (Phipps *et al*., 2019). We recognise that the convenience sample introduces a potential source of bias. Despite the inherent limitations associated with this method, we implemented targeted efforts to mitigate potential bias. This included establishing partnerships with local community organisations and healthcare providers, collaborating with outreach programmes and employing culturally sensitive recruitment strategies.

Thirdly, earlier research has demonstrated inconsistent findings regarding the reliability of self-reported measures of HL compared to objectively assessed HL (Smith and Haggerty, 2003; Farrell *et al*., 2020). Finally, the cross-sectional design limits inferences of causality or temporality regarding HL and well-being. Longitudinal studies are needed to investigate stability and change in associations between HL and well-being among women experiencing homelessness.

## CONCLUSION

The present study provides empirical evidence on the association between HL and well-being among women experiencing homelessness. A higher degree of HL was found to be associated with both lower psychological distress and higher spiritual well-being among women experiencing homelessness. Practical applications of this study include the development of targeted and tailored HL interventions for women experiencing homelessness, focusing on both mental and spiritual well-being. This group is disproportionately impacted by the burden of disease and disability, and women experiencing homelessness face substantial barriers to accessing health information and services. Thus, a proportionate universalist approach may be adopted to ensure that health promotion interventions are as accessible and usable as possible for all of society, whilst providing tailored interventions to promote HL among women experiencing homelessness, which may enhance their mental and spiritual well-being. Crucially, the development of tailored interventions should involve end users with lived experiences of homelessness, and further research is needed regarding the co-production of health promotion interventions with women experiencing homelessness. In terms of tailored interventions, the Women’s Advisory Board for Inclusion Health emphasised that a ‘housing first’ approach is needed with adequate support to address their basic needs, and HL promotion initiatives may contribute to this targeted support for women experiencing homelessness.

## Supplementary Material

daae019_suppl_Supplementary_Material
